# Six years’ accomplishment of the Initiative on Rare and Undiagnosed Diseases: nationwide project in Japan to discover causes, mechanisms, and cures

**DOI:** 10.1038/s10038-022-01025-0

**Published:** 2022-03-23

**Authors:** Yuji Takahashi, Hidetoshi Date, Hideki Oi, Takeya Adachi, Noriaki Imanishi, En Kimura, Hotake Takizawa, Shinji Kosugi, Naomichi Matsumoto, Kenjiro Kosaki, Yoichi Matsubara, IRUD Consortium, Yukio Ando, Toshihisa Anzai, Tadashi Ariga, Yoshimitsu Fukushima, Yoshihiko Furusawa, Akira Ganaha, Yuichi Goto, Kenichiro Hata, Masataka Honda, Kazumoto Iijima, Tsunakuni Ikka, Issei Imoto, Tadashi Kaname, Masao Kobayashi, Seiji Kojima, Hiroki Kurahashi, Shigeo Kure, Kenji Kurosawa, Yoshihiro Maegaki, Yoshio Makita, Tomohiro Morio, Ichiei Narita, Fumio Nomura, Tsutomu Ogata, Keiichi Ozono, Akira Oka, Nobuhiko Okamoto, Shinji Saitoh, Akihiro Sakurai, Fumio Takada, Tsutomu Takahashi, Akira Tamaoka, Akihiro Umezawa, Akihiro Yachie, Kouichiro Yoshiura, Yasutsugu Chinen, Mariko Eguchi, Keishi Fujio, Kiminori Hosoda, Tomohiko Ichikawa, Toshitaka Kawarai, Tomoki Kosho, Mitsuo Masuno, Akie Nakamura, Takaya Nakane, Tomoo Ogi, Satoshi Okada, Yasushi Sakata, Toshiyuki Seto, Yoshiyuki Takahashi, Tadao Takano, Mitsuharu Ueda, Hideaki Yagasaki, Toshiyuki Yamamoto, Atsushi Watanabe, Yoshihiro Hotta, Akiharu Kubo, Hirofumi Maruyama, Keiji Moriyama, Eiji Nanba, Norio Sakai, Yoshiki Sekijima, Toru Shimosegawa, Tsutomu Takeuchi, Shinichi Usami, Kazuhiko Yamamoto, Hidehiro Mizusawa

**Affiliations:** 1 Department of Neurology, National Center Hospital, National Center of Neurology and Psychiatry, Kodaira, Japan; 2 Department of Clinical Data Science, Clinical Research and Education Promotion Division, National Center of Neurology and Psychiatry, Kodaira, Japan; 3 Keio Frontier Research & Education Collaborative Square (K-FRECS) at Tonomachi, Keio University, Kawasaki, Japan; 4 Department of Medical Regulatory Science, Kyoto Prefectural University of Medicine, Graduate School of Medical Science, Kyoto, Japan; 5 Japan Agency for Medical Research and Development (AMED), Tokyo, Japan; 6 Department of Research Promotion and Management, National Cerebral and Cardiovascular Center, Suita, Japan; 7 Astellas Pharma Incorporated, Tokyo, Japan; 8 Department of Medical Ethics/Medical Genetics, Kyoto University School of Public Health, Kyoto, Japan; 9 Department of Human Genetics, Yokohama City University Graduate School of Medicine, Yokohama, Japan; 10 Center for Medical Genetics, Keio University School of Medicine, Tokyo, Japan; 11 National Center for Child Health and Development, Tokyo, Japan; 12 Department of Neurology, Graduate School of Medical Sciences, Kumamoto University, Kumamoto, Japan; 13 Department of Amyloidosis Research, Nagasaki International University, Nagasaki, Japan; 14 Department of Cardiovascular Medicine, National Cerebral and Cardiovascular Center, Suita, Japan; 15 Department of Cardiovascular Medicine, Hokkaido University Graduate School of Medicine, Sapporo, Japan; 16 Department of Pediatrics, Hokkaido University Hospital, Sapporo, Japan; 17 Department of Medical Genetics, Shinshu University School of Medicine, Matsumoto, Japan; 18 Japan Medical Office, Takeda Pharmaceutical Company Limited, Tokyo, Japan; 19 Department of Otorhinolaryngology, Head and Neck Surgery, Graduate School of Medicine, University of the Ryukyus, Nishihara, Japan; 20 Department of Otolaryngology, Faculty of Medicine, University of Miyazaki, Miyazaki, Japan; 21 Medical Genome Center, National Center of Neurology and Psychiatry, Kodaira, Japan; 22 Department of Maternal-Fetal Biology, National Research Institute for Child Health and Development, Tokyo, Japan; 23 Department of Nephrology, Tokyo Metropolitan Children’s Medical Center, Fuchu, Japan; 24 Department of Pediatrics, Kobe University Graduate School of Medicine, Kobe, Japan; 25 Translational Medical Center, National Center of Neurology and Psychiatry, Kodaira, Japan; 26 Division of Bioethics and Healthcare Law, Center for Public Health Sciences, National Cancer Center, Tokyo, Japan; 27 Department of Human Genetics, Graduate School of Biomedical Science, Tokushima University Graduate School, Tokushima, Japan; 28 Division of Molecular Genetics, Aichi Cancer Center Research Institute, Nagoya, Japan; 29 Department of Pediatrics, Hiroshima University Graduate School of Biomedical and Health Sciences, Hiroshima, Japan; 30 Department of Pediatrics, Nagoya University Graduate School of Medicine, Nagoya, Japan; 31 Division of Molecular Genetics, Institute for Comprehensive Medical Science, Fujita Health University, Toyoake, Japan; 32 Department of Pediatrics, Tohoku University Graduate School of Medicine, Sendai, Japan; 33 Division of Medical Genetics, Kanagawa Children’s Medical Center, Yokohama, Japan; 34 Division of Child Neurology, Department of Brain and Neurosciences, Faculty of Medicine, Tottori University, Yonago, Japan; 35 Department of Genetic Counseling, Asahikawa Medical University Hospital, Asahikawa, Japan; 36 Department of Pediatrics and Developmental Biology, Graduate School of Medical and Dental Sciences, Tokyo Medical and Dental University (TMDU), Tokyo, Japan; 37 Division of Clinical Nephrology and Rheumatology, Niigata University Graduate School of Medical and Dental Sciences, Niigata, Japan; 38 Division of Clinical Mass Spectrometry, Chiba University Hospital, Chiba, Japan; 39 Department of Pediatrics, Hamamatsu University School of Medicine, Hamamatsu, Japan; 40 Department of Pediatrics, Osaka University Graduate School of Medicine, Suita, Japan; 41 Department of Pediatrics, Graduate School of Medicine, The University of Tokyo, Tokyo, Japan; 42 Division of Neurology, Saitama Children’s Medical Center, Saitama, Japan; 43 Department of Medical Genetics, Osaka Women’s and Children’s Hospital, Izumi, Japan; 44 Department of Pediatrics and Neonatology, Nagoya City University Graduate School of Medical Sciences, Nagoya, Japan; 45 Department of Medical Genetics and Genomics, School of Medicine, Sapporo Medical University, Sapporo, Japan; 46 Department of Medical Genetics and Genomics, Kitasato University Graduate School of Medical Sciences, Sagamihara, Japan; 47 Department of Pediatrics, Akita University Graduate School of Medicine, Akita, Japan; 48 Department of Neurology, Faculty of Medicine, University of Tsukuba, Tsukuba, Japan; 49 Department of Reproductive Biology, National Research Institute for Child Health and Development, Tokyo, Japan; 50 Division of Medical Safety, Kanazawa University Hospital, Kanazawa, Japan; 51 Department of Human Genetics, Atomic Bomb Disease Institute, Nagasaki University, Nagasaki, Japan; 52 Department of Pediatrics, Faculty of Medicine, University of the Ryukyus, Nishihara, Japan; 53 Department of Pediatrics, Ehime University Graduate School of Medicine, Matsuyama, Japan; 54 Department of Allergy and Rheumatology, Graduation School of Medicine, The University of Tokyo, Tokyo, Japan; 55 Department of Diabetes and Lipid Metabolism, National Cerebral and Cardiovascular Center, Suita, Japan; 56 Departments of Urology, Chiba University Graduate School of Medicine, Chiba, Japan; 57 Department of Neurology, Hyogo Brain and Heart Center, Himeji, Japan; 58 Department of Clinical Neuroscience, Institute of Biomedical Sciences, Graduate School of Medical Sciences, Tokushima University, Tokushima, Japan; 59 Genetic Counseling Program, Graduate School of Health and Welfare, Kawasaki University of Medical Welfare, Kurashiki, Japan; 60 Department of Pediatrics, Hokkaido University School of Medicine, Sapporo, Japan; 61 Department of Pediatrics, Faculty of Medicine, University of Yamanashi, Chuo, Japan; 62 Department of Genetics, Research Institute of Environmental Medicine (RIeM), Nagoya University, Nagoya, Japan; 63 Department of Human Genetics and Molecular Biology, Nagoya University, Nagoya, Japan; 64 Department of Cardiovascular Medicine, Osaka University Graduate School of Medicine, Suita, Japan; 65 Medical Genetics, Graduate School of Medicine, Osaka City University, Osaka, Japan; 66 Clinical Research, Innovation and Education Center, Tohoku University Hospital (CRIETO), Sendai, Japan; 67 Center of Genetic Medicine, Hospital, University of Yamanashi, Chuo, Japan; 68 Institute of Medical Genetics, Tokyo Women’s Medical University, Tokyo, Japan; 69 Division of Clinical Genetics, Kanazawa University Hospital, Kanazawa, Japan; 70 Department of Ophthalmology, Hamamatsu University School of Medicine, Hamamatsu, Japan; 71 Department of Dermatology, Keio University School of Medicine, Tokyo, Japan; 72 Division of Dermatology, Department of Internal Related, Kobe University Graduate School of Medicine, Kobe, Japan; 73 Department of Clinical Neuroscience and Therapeutics, Graduate School of Biomedical and Health Sciences, Hiroshima University, Hiroshima, Japan; 74 Department of Maxillofacial Orthognathics, Tokyo Medical and Dental University, Graduate School, Tokyo, Japan; 75 Division of Functional Genomics, Research Center for Bioscience and Technology, Tottori University, Yonago, Japan; 76 Department of Medicine (Neurology and Rheumatology), Shinshu University School of Medicine, Matsumoto, Japan; 77 Division of Gastroenterology, Tohoku University Graduate School of Medicine, Sendai, Japan; 78 Keio University School of Medicine, Keio University Hospital, Tokyo, Japan; 79 Department of Otorhinolaryngology, Shinshu University School of Medicine, Matsumoto, Japan; 80 Department of Allergy and Rheumatology, Graduate School of Medicine, The University of Tokyo, Tokyo, Japan; 81 Laboratory for Autoimmune Diseases, Center for Integrative Medical Sciences, RIKEN, Yokohama, Japan

**Keywords:** Disease genetics, Genetics research

## Abstract

The identification of causative genetic variants for hereditary diseases has revolutionized clinical medicine and an extensive collaborative framework with international cooperation has become a global trend to understand rare disorders. The Initiative on Rare and Undiagnosed Diseases (IRUD) was established in Japan to provide accurate diagnosis, discover causes, and ultimately provide cures for rare and undiagnosed diseases. The fundamental IRUD system consists of three pillars: IRUD diagnostic coordination, analysis centers (IRUD-ACs), and a data center (IRUD-DC). IRUD diagnostic coordination consists of clinical centers (IRUD-CLs) and clinical specialty subgroups (IRUD-CSSs). In addition, the IRUD coordinating center (IRUD-CC) manages the entire IRUD system and temporarily operates the IRUD resource center (IRUD-RC). By the end of March 2021, 6301 pedigrees consisting of 18,136 individuals were registered in the IRUD. The whole-exome sequencing method was completed in 5136 pedigrees, and a final diagnosis was established in 2247 pedigrees (43.8%). The total number of aberrated genes and pathogenic variants was 657 and 1718, among which 1113 (64.8%) were novel. In addition, 39 novel disease entities or phenotypes with 41 aberrated genes were identified. The 6-year endeavor of IRUD has been an overwhelming success, establishing an all-Japan comprehensive diagnostic and research system covering all geographic areas and clinical specialties/subspecialties. IRUD has accurately diagnosed diseases, identified novel aberrated genes or disease entities, discovered many candidate genes, and enriched phenotypic and pathogenic variant databases. Further promotion of the IRUD is essential for determining causes and developing cures for rare and undiagnosed diseases.

## Introduction

Identification of the causative genetic variants of hereditary diseases has revolutionized clinical medicine to enhance diagnostic accuracy, understand disease pathogenesis, and develop therapies. The major technical breakthrough behind the revolution was the development of whole-genome sequencing (WGS) and whole-exome sequencing (WES) methods employing next-generation sequencing (NGS), enabling identification of causative genetic variants by simultaneously capturing all candidate variants potentially causing diseases in affected individuals. Additionally, tremendous amounts of variant data have been generated using sequencers and remarkable advances have been made in bioinformatics exploiting such big data with powerful computational data analysis methods. Accumulation of variant data via registration in public databases has also accelerated the discovery of pathogenic variants by filtering known variants that are not associated with diseases.

However, there still remain many diseases for which causative genetic variants have not been identified. According to Online Mendelian Inheritance in Man (OMIM) (URL: https://www.omim.org/), 9514 hereditary diseases were registered as of November 2021, of which 3288 diseases with (suspected) Mendelian basis are classified as having an unknown molecular basis. A complete understanding of the molecular basis of these diseases is one of the ultimate goals of human molecular genetics, which remains highly challenging even in the NGS era. The difficulty lies in identifying multiple pedigrees with pathogenic variants in the same genes to verify causality, particularly when researchers attempt to discover causative genetic variants for ultra-rare diseases, which are thought to comprise most unsolved diseases [1]. Even when a novel candidate gene is identified in a given pedigree with an ultra-rare disorder, it is almost impossible to discover a second pedigree within a single institute or a single consortium, known as the N-of-1 problem. Extensive data sharing across multiple institutions and international collaboration is key to overcoming this difficulty.

To solve the N-of-1 problem, the formation of extensive collaborative frameworks with international cooperation has become a global trend [2]. The Undiagnosed Diseases Network in the USA [1], Genomics England in England [3] and Finding of Rare Disease Genes in Canada [4] are the three leading projects that have achieved unprecedented success in identifying causative genetic variants of many rare disorders. In 2011, the International Rare Disease Research Consortium (IRDiRC) (https://irdirc.org/about-us/history/) established a worldwide network to connect individual projects to conduct international collaborative studies, further promoting the discovery of causative genetic variants [5]. In 2015, the Agency for Medical and Research Development (AMED) participated in IRDiRC as the first organization from Japan [6] and set specific action goals to promote international collaboration through further data sharing to contribute to the AMED-affiliated IRDiRC 10-year plan [7]. Furthermore, Undiagnosed Disease Network International was established on 2015 to build a consensus framework of principles, best practice, and governance involving these projects [8]. One of the key factors behind the success of these projects has been the development of the MatchMaker Exchange (MME), an extensive data-sharing system connecting genomic and phenotypic databases based on a unified computational architecture and common application programming interface [9]. MME adopts human phenotype ontology (HPO) as the standardized language to describe phenotypes [10] and facilitates the computation of phonotype and genotype matching to identify multiple pedigrees with the same aberrated genes.

Even when causative genetic variants have been established, genetic diagnosis of rare diseases remains difficult in clinical settings. One reason for this is that a limited number of patients undergo WGS- or WES-based genetic diagnosis. Accessibility is among the key factors that can drive the utilization of this innovative technology in clinical genetics, necessitating a nationwide infrastructure to regionally equalize this testing. Additionally, determining pathogenic variants among a large number of variants yielded by WGS/WES and establishing the final diagnosis in which the pathogenic variants fully account for clinical manifestations is labor-intensive. Particularly, a substantial number of rare and undiagnosed diseases present with complex phenotypes with multiple affected organs, making the determination difficult by researchers in a single specialty. Therefore, it is recommended that diagnostic boards composed of physicians with a wide range of specialties and geneticists should discuss the final diagnosis based on the phenotypes and WGS/WES data.

Enhancing the diagnostic accuracy of rare diseases has been vigorously pursued in Japan. Remarkable achievements have been made in the research and countermeasures for rare and intractable diseases, designated as “Nan-byo,” by the Ministry of Health, Labor and Welfare in Japan, which was established in 1972 after the subacute myelo-optic neuropathy endemic. In 2015, a new intractable disease law was enforced to expand “Nan-byo” from 56 to 333 diseases, further promoting the diagnosis of rare and intractable diseases. Nevertheless, two surveys conducted by AMED showed that more than 37,000 cases remained undiagnosed [11].

To address these issues, the Initiative on Rare and Undiagnosed Diseases (IRUD) was launched in 2015 as a nationwide project in Japan supported by AMED [6]. IRUD aims to establish accurate diagnoses, discover causes, and ultimately provide cures for diseases through nationwide coverage of comprehensive diagnostic systems, utilization of innovative tests including NGS, and construction of an internationally sharable clinical database [11]. Initially, IRUD for pediatric patients (IRUD-P) was launched in July 2015, followed by IRUD for adult patients (IRUD-A) in October. In 2017, the two were integrated into one project as the IRUD to make the project more extensive and comprehensive. This study describes the accomplishments of the 6-year effort of the IRUD project, illustrating the whole diagnostic system, diagnostic yield and pathogenic variant landscape of rare and undiagnosed diseases, novel genes/disease entities, and human resource development.

## Materials and methods

### IRUD entry criteria

The IRUD entry criteria are as follows [6].

1. The patient remains undiagnosed for ≥6 months (not necessary for infants) and suffers from disabilities in daily life, AND

2–1. Objective signs exist that cannot be attributed to a single organ; OR

2–2. Direct or indirect evidence exists of a genetic etiology (e.g. similar symptom(s) found in the patient’s relatives) [6].

Here, an undiagnosed disease is clearly distinguished from an undetermined disease in which a clinical diagnosis has been made but its causative genetic variants have not been confirmed. For example, if spinocerebellar degeneration is clinically diagnosed, although its causative genetic variants have not been analyzed and disease type has not been determined, it is classified as an undetermined disease and excluded from the IRUD. This clearly distinguishes the IRUD from genetic diagnostic services.

### Genomic analysis

Blood samples were obtained from the participants fulfilling the criteria mentioned above with informed consent, DNA samples were extracted, and B-cell lymphoblast cell lines were established. Genomic DNA was subjected to enrichment of exonic sequences using Agilent SureSelect (Agilent Technologies, Santa Clara, CA, USA). Massively parallel sequencing (100-base pair paired-end reads) was performed using NGS (Hiseq2500; Illumina, San Diego, CA, USA). The sequences were aligned to the reference genome (GRCh37/hg19) using the Burrows-Wheeler Aligner or NovoAlign. Removal of potential polymerase chain reaction duplicates, recalibration of base quality values, local realignment, and variation calls were performed using SAMtools, Picard, and GATK with default parameter settings for alignment of raw reads and detection of single-nucleotide variants and short insertion/deletion variants. These variants were annotated using ANNOVAR (https://annovar.openbioinformatics.org/) together with RefSeq (http://www.ncbi.nlm.nih.gov/RefSeq/), 1000 Genomes Project Database (http://www.1000genomes.org/), dbSNP135 (http://www.ncbi.nlm.nih.gov/projects/SNP/), gnomAD (http://www.gnomad.broadinstitute.org), and in-house databases.

### Data sharing

A data-sharing platform, IRUD Exchange, was designed by incorporating the Patient Archive system [12] that complies with HPO and can be linked with the MME. The architecture of the IRUD Exchange allows IRUD researchers to conduct similarity searches using pattern-matching algorithms as a powerful tool to address ‘N-of-1’ problems of rare diseases. The IRUD Exchange also facilitates the registration of HPO-based phenotypes by adopting a user-friendly interface that automatically translates clinical summaries written in Japanese into English and highlights relevant HPO terms.

### Central ethics committee

Initially, the IRUD-P started with approval from the individual institutional ethics committee. Subsequently, a central ethics committee (CEC) was established in IRUD-A as one of the leading model projects in AMED to facilitate the ethical review process in multi-institutional large-scale collaborative research. All but one of the individual institutional ethical committees in IRUD-A delegated the reviewing process to the CEC. The delegation process had been further promoted upon the integration of IRUD-A and IRUD-P.

A unified research protocol for IRUD was reviewed and approved by the CEC. The ethics committee of individual institutions delegated the review process to the CEC, where approval of the protocol allowed each institute to initiate IRUD research based on the unified protocol. Amendment of the unified protocol, such as authentication of newly participating institutes, is accomplished in a one-step review process as an entire IRUD project. This study was approved by the CEC at Tohoku University on February 20, 2018 (CEC No. 2017-2-303).

## Results

### IRUD diagnostic and research system

**Fig. 1:**
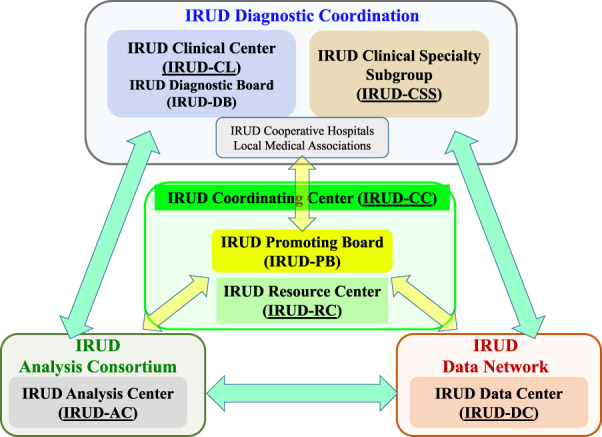
Initiative on Rare and Undiagnosed Diseases (IRUD) diagnostic and research system. IRUD diagnostic and research system consists of six components indicated by underlines: IRUD Clinical Center (IRUD-CL), IRUD Clinical Specialists Subgroup (IRUD-CSS), IRUD Data Center (IRUD-DC), IRUD Analysis Center (IRUD-AC), IRUD Resource Center (IRUD-RC), and IRUD Coordination Center (IRUD-CC). IRUD-CC manages the IRUD Promoting Board (IRUD-PB), the highest decision-making organization. IRUD-CL manages the IRUD Diagnostic Board (IRUD-DB), which manages the process from the decision on patient entry to establishment of a final diagnosis. IRUD-CL and IRUD-CSS are integrated into IRUD Diagnostic Coordination, in which community clinics/hospitals belonging to the Local Medical Association participate. IRUD-CC also temporally serves as an IRUD-RC

The most important achievement of IRUD is establishment of a unified all-Japan diagnostic and research system for rare and undiagnosed diseases covering entire geographic areas and clinical specialty/subspecialty fields. The IRUD system consists of three pillars: IRUD diagnostic coordination, analysis centers (IRUD-ACs), and a data center (IRUD-DC). IRUD diagnostic coordination consists of clinical centers (IRUD-CLs) and clinical specialty subgroups (IRUD-CSSs). In addition, the IRUD coordinating center (IRUD-CC) manages the entire IRUD system and temporarily operates the IRUD Resource Center (IRUD-RC) (Fig. 1).

#### IRUD Coordination Center (IRUD-CC)

The principal role of IRUD-CC is administration of the whole system through monthly IRUD-PB meetings as the highest decision-making organization. The constituents of IRUD-PB include representatives of the IRUD-CC (principal investigator), AMED (program officers) as the funding agency, IRUD-CLs, IRUD-CSSs, IRUD-ACs, and IRUD-DCs. IRUD-CC drafts a unified research protocol that is ratified by IRUD-PB and subjected to CEC, with the one-step approval process contributing to timely modification of the IRUD research. In addition, IRUD-CC monitors the progress of the entire research by conducting a regular survey. IRUD-CC also operates the sample and information logistics system described in detail in a subsequent section.

#### IRUD Clinical Center (IRUD-CL) and Semi-Clinical Center (IRUD-SCL)

IRUD-CL/SCL is the only contact site for patients with IRUD. Upon patient entry, parents are recruited so that trio analysis can be performed. IRUD-CL/SCL operates the IRUD Diagnostic Board (IRUD-DB), which manages the process from patient entry decisions to final diagnosis establishment. IRUD-SCLs are designed to fill the geographic gaps of IRUD-CLs, although no funding was provided. IRUD Cooperative Hospitals refer candidates for IRUD entry to IRUD-CL/IRUD-SCL. The IRUD-DB plays a central role in the regional diagnostic network with IRUD Cooperative Hospitals. The IRUD-DB is composed of pediatricians and physicians of various specialties/subspecialties for adults, clinical geneticists, genetic counselors, and data scientists. The IRUD protocol stipulates the participation of representative physicians from local medical associations in each IRUD-DB to promote regional cooperation.

IRUD-DB holds regular meetings during which thorough pre-entry evaluation is conducted based on the clinical information described in a regular format on a ‘patient sheet’ to determine whether the candidate is suitable for IRUD entry and if sufficient investigation has already been completed, including whether clinical workups and available genetic tests such as chromosome analysis or gene-panel analysis have been performed. Similarly, post-analysis evaluation is conducted at the regular meetings to determine whether the pathogenic variant reported by IRUD-AC fully accounts for the clinical phenotypes leading to the final clinical diagnosis. In addition, the activities of the IRUD-DB include genetic counseling, further follow-up and reevaluation of the pedigree, public relations, and human resource development. Thus, IRUD-CL/SCL plays an essential role in the clinical aspects of the IRUD system.

#### IRUD Clinical Specialty/Subspecialty Subgroup (IRUD-CSS)

IRUD-CSSs are organized by assembling members of IRUD-DBs across entire IRUD-CLs according to their specialties/subspecialties. IRUD-CSSs support the activities of individual IRUD-DBs and provide professional advice based on their specialties/subspecialties for cases that cannot be resolved by IRUD-CLs alone.

Thus, IRUD-CLs and IRUD-CSSs form the IRUD Diagnostic Coordination covering entire geographic areas and specialty/subspecialty fields in Japan.

#### IRUD Analysis Center (IRUD-AC)

Each IRUD-CL/IRUD-SCL sends DNA samples via an outsourcing provider to a corresponding single IRUD-AC, which conducts comprehensive genomic analysis, identifies pathogenic variants, and reports to the IRUD-CL/IRUD-SCL via the IRUD-CC. When pathogenic variants are undetermined, further intensive research is conducted to identify novel aberrated genes/pathogenic variants using WGS, multi-omics analysis, and functional studies.

#### IRUD Data Center (IRUD-DC)

IRUD-DC operates the IRUD Exchange, the data-sharing platform described above, promotes data sharing among IRUD researchers, and serves as a gateway to domestic and international collaboration. Phenotypic and genomic information has been accumulated to promote the establishment of new causative genetic variants and disease concepts. The IRUD Exchange is also used as a database to understand the overall epidemiological landscape of rare and undiagnosed diseases registered in the IRUD. All IRUD-CLs and IRUD-SCLs have a computer terminal for the IRUD-Exchange, and phenotypic and genotype data are transferred to IRUD-DC through a specific virtual private network to ensure security.

#### IRUD Resource Center (IRUD-RC)

IRUD-RC establishes a resource repository for clinical information and DNA samples/B cell lines and manages a utilization committee for examining the utilization of repositories. The IRUD-CC temporarily serves as the IRUD-RC, which is planned to be established as an independent facility assigned by AMED.

### IRUD workflow of samples/information

**Fig. 2:**
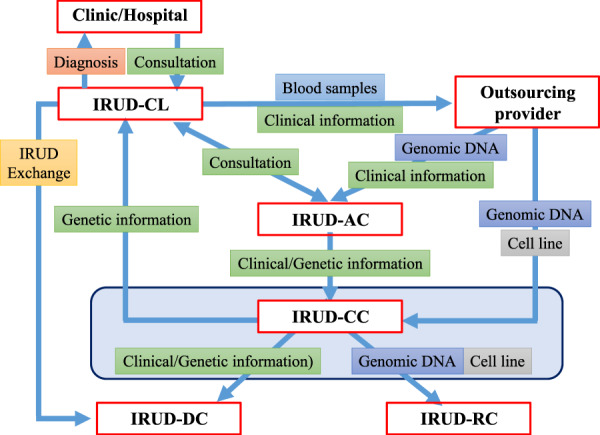
Sample/information workflow. Initiative on Rare and Clinical Diseases Coordination Center (IRUD-CC) manages a sample/information workflow using a unified identification number system. The workflow utilizes an outsourcing provider to extract genomic DNA samples and establish B lymphoblast cell lines, facilitating the flow of samples and information from IRUD Clinical Centers (IRUD-CLs) to IRUD Analysis Centers (IRUD-ACs) and centralizing information and sample repositories in the IRUD Resource Center (IRUD-RC)

IRUD-CC organizes a unified workflow to facilitate sample and information sharing among the IRUD-CLs/IRUD-SCLs, IRUD-ACs, and IRUD-CC and establishes a centralized repository in IRUD-RC (Fig. 2).

Upon entry into the IRUD, an individual identification (ID) number, composed of a three-alphabetical institutional code and five-digit numerical number, is allotted to each registrant, whose DNA/cell line samples, clinical information, and analysis results are handled with a specific ID. The workflow utilizes an outsourcing provider to extract genomic DNA samples and establish B lymphoblast cell lines. The genomic DNA samples are sent to the IRUD-ACs and IRUD-CC, and B lymphoblast cell lines are sent to the IRUD-CC. Each IRUD-CL/IRUD-SCL sends the samples to a specific IRUD-AC designated by IRUD-CC. Clinical information in the form of a ‘patient sheet’ is also sent to IRUD-ACs via an outsourcing provider. The analysis results and clinical information are sent from the IRUD-ACs to the IRUD-CC and reported to the corresponding IRUD-CLs. Clinical information is accumulated in the IRUD-DC through IRUD-Exchange from the IRUD-CLs. Text-based clinical data on the patient sheet delivered from the IRUD-CL/SCL via the IRUD-AC and analysis reports delivered from the IRUD-AC are also stored in the IRUD-CC. Genomic DNA samples and B lymphoblast cell lines are deposited in the IRUD-RC.

### Present status and activities of the IRUD system

**Fig. 3:**
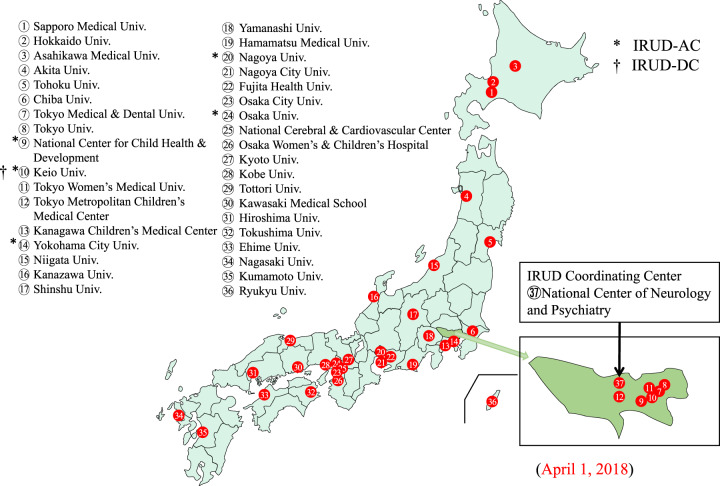
Location of Initiative on Rare and Clinical Diseases Clinical Center (IRUD-CLs) since fiscal year 2018. Locations of each IRUD-CL are shown in the corresponding numbers. The National Center of Neurology and Psychiatry also operates as an IRUD Coordinating Center (IRUD-CC). The IRUD-CLs operating as IRUD Analysis Center (IRUD-AC) are shown with asterisks, and an IRUD-CL operates as an IRUD Data Center (IRUD-DC) and is indicated with a dagger. Inset: IRUD CLs in Tokyo Metropolis. Univ.: University

In March 2021, the IRUD diagnostic system comprised 450 institutions consisting of 37 IRUD-CLs, 15 IRUD-SCLs, and 398 cooperative hospitals (Fig. 3). Five IRUD-CLs also serve as IRUD-ACs, one of which also serves as the IRUD-DC. The National Center of Neurology and Psychiatry serves as the IRUD-CC, IRUD-CL, and IRUD-RC. Twenty-one IRUD-CSSs included 497 clinical specialists to support IRUD-DBs in the IRUD-CLs (Table 1). The IRUD-RC established resource repositories, including 4489 genomic DNA samples and 3017 lymphoblastic cell lines. Phenotypes and genetic data of 5378 pedigrees have been registered on the IRUD Exchange, among which 62 are shared internationally through the MME. Thirty-two IRUD-CLs have delegated the ethics review process to CEC, the remaining 5 utilize their own institutional review boards.

**Table 1:** Clinical specialist/subspecialist subgroups (IRUD-CSSs)

Category	Subcategory	Number	Category	Number
Pediatrics	General	65	Allergy/Rheumatology	19
	Metabolic diseases	5	Immunodeficiency	11
	Congenital abnormality	12	Orthopedics	17
Obstetrics		44	Dermatology	20
Neurology		50	Ophthalmology	18
Pulmonology		5	Otorhinolaryngology	26
Cardiology		26	Dentistry	10
Gastroenterology		32	Psychiatry	12
Nephrology/Urology		27	Clinical genetics	50
Endocrinology		30	Local medical association	24
Hematology		21	Total	524

In November 2021, 21 IRUD-CSSs were organized to actively support Initiative on Rare and Clinical Diseases Diagnostic Boards (IRUD-DBs). The number of members in each IRUD-CSS is shown

### Diagnostic yield and pathogenic variant landscape

**Fig. 4:**
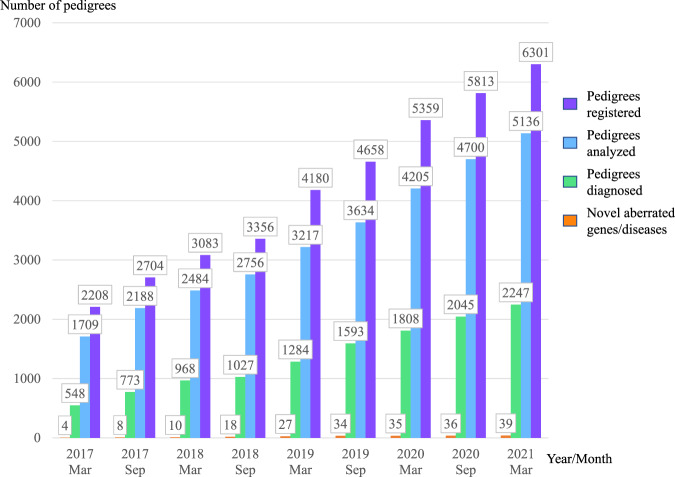
Initiative on Rare and Clinical Diseases (IRUD) diagnostic yield. The cumulative number of pedigrees entered, pedigrees with analysis completed, pedigrees with diagnosis established, and novel genes or disease entities are shown at the surveyed year and month

**Fig. 5:**
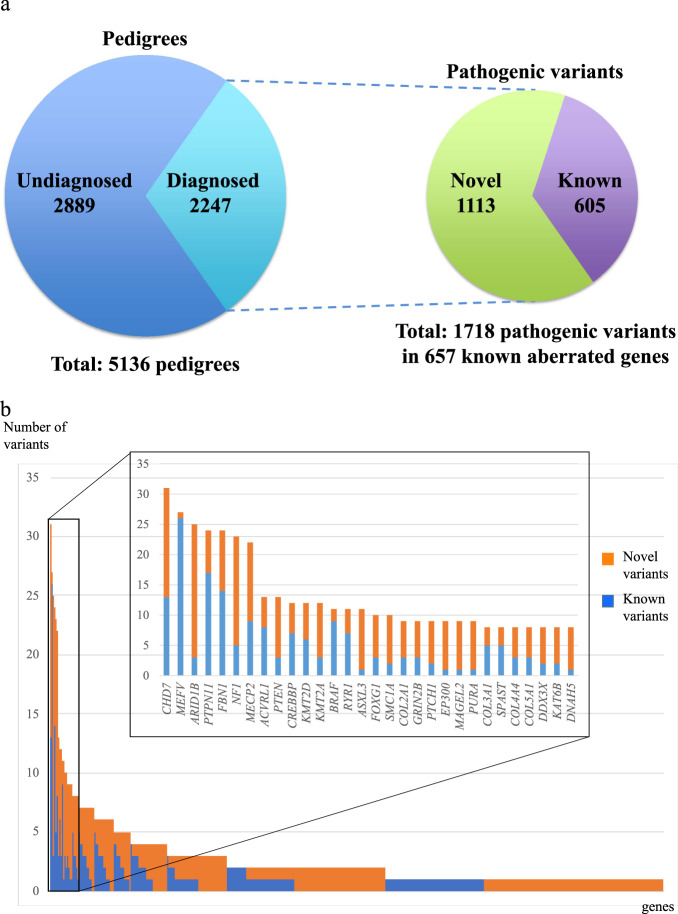
Pathogenic variant landscape in the Initiative on Rare and Clinical Diseases (IRUD). **a** Breakdown of pathogenic variants. Diagnosis was established in 2247of 5136 pedigrees. A total of 1718 pathogenic variants were identified in 657 known aberrated genes, among which 1113 were novel (64.8%). **b** Overview of the frequencies of pathogenic variants in each gene. The number of novel pathogenic variants (shown in blue) and that of known pathogenic variants (shown in orange) in individual genes were arranged in the order of the total number of pathogenic variants. The most frequent gene was *CHD7* with 31 pathogenic variants, whereas 298 of the genes had only one pathogenic variant. Inset: genes identified in >7 pedigrees

In March 2021, 6301 pedigrees consisting of 18136 individuals were registered in the IRUD. WES was completed in 5136 pedigrees, with a final diagnosis established in 2247 pedigrees (43.8%) (Fig. 4). The total numbers of aberrated genes and pathogenic variants in these pedigrees were 657 and 1718, respectively; 1113 (64.8%) of the variants were novel (Fig. 5a). Among the 2247 pedigrees, the most frequently identified causative gene was *CHD7*, which was identified in 31 pedigrees, followed by *MEFV* in 27 pedigrees and *ARID1B* in 25 pedigrees. In contrast, 298 aberrated genes were causative in single pedigrees, exhibiting a long-tail distribution of gene frequencies (Fig. 5b) (Supplementary Table 1). Most pathogenic variants are unique, whereas some have been identified in multiple pedigrees. Notably, a known pathogenic variant p.E148Q in *MEFV* and known p.N308S mutation in *PTPN11* were identified in eight and five pedigrees, respectively, making these pathogenic variants relatively common among undiagnosed diseases in the Japanese population.

### Novel aberrated genes or disease entities

Thirty-nine novel disease entities or phenotypes along with 41 aberrated genes were identified in the IRUD (Fig. 4, Supplementary Table 2). These genes were classified into three categories. Category 1: Novel disease entities with novel aberrated genes. Category 2: Novel disease entities to which unique OMIM IDs are assigned to novel pathogenic variants in known aberrated genes. Category 3: Novel phenotypes in known disease entities with novel pathogenic variants of known aberrated genes. Category 1 comprised 23 disease entities with 24 genes [13–32], category 2 comprised 4 disease entities with 4 genes [33–36], and category 3 comprised 12 disease entities with 13 genes [37–47]. In addition, among the remaining 2889 families in whom causative genetic variants had not been established, 313 pedigrees belonged to the N-of-1 category, in which only one candidate gene per pedigree was determined by WES but remained as a single family, awaiting further discovery of additional pedigrees.

### Human resource development

The IRUD offers an outstanding opportunity for human resource development through on-the-job training. In fiscal years 2018, 2019, and 2020, there were 78, 87, and 24 IRUD members and related people who received promotions in their institutes; 26, 34, and 0 were promoted in other institutes; 12, 47, and 8 became staff inside their institutes; 10, 5, and 0 were given jobs in companies associated with clinical genetics; 56, 62, and 38 achieved certification by the Japanese Board of Medical Genetics and Genomics, Clinical Genetics; and 17, 50, and 12 obtained certification as Genetic Counselors, respectively. IRUD has also contributed to the training of data scientists: 64, 65, and 5 medical doctors and 32, 38, and 4 non-medical doctors participated in genome informatics analysis as data scientists in 2018, 2019, and 2020, respectively.

## Discussion

The 6-year endeavor of the IRUD has been an overwhelming success, establishing an all-Japan comprehensive diagnostic and research system for rare and undiagnosed diseases covering entire geographic areas and clinical specialties/subspecialties. The IRUD has led to the accurate diagnoses, identification of novel aberrated genes or disease entities, discovery of many candidate genes, enrichment of phenotypic and pathogenic variant databases, and development of treatments and cures. It also has established a fundamental infrastructure for both centralized governance with unified protocols, logistics, biorepositories, data sharing and ethics and individual autonomous research activities among the IRUD-AC, IRUD-DC, and IRUD-CL/SCL. Thus, the IRUD is a unified, sustainable medical and research system that can be expanded to all fields of genomic medicine.

The IRUD has established an accurate diagnosis for a large number of patients and, in some cases, led to the use of specific therapies with positive effects [40]. Pathogenic variants have been identified in 657 genes, encompassing more than one-tenth of all aberrated genes registered in the OMIM. The diagnostic yield is 43.8% (2247 in 5136 pedigrees), which is comparable to that of the Undiagnosed Diseases Network (30%: 427 in 1413 pedigrees) (https://undiagnosed.hms.harvard.edu/about-us/facts-and-figures/). Particularly, approximately one-half of pedigrees with an established diagnosis possess unique aberrated genes. Importantly, such diseases are individually ‘ultra-rare’ but not as a whole, necessitating further vigorous endeavors to provide accurate diagnosis and develop therapeutic measures for individual ‘ultra-rare’ diseases.

The IRUD has had important impacts on basic research by identifying many novel aberrated genes, establishing novel disease entities, and detecting novel pathogenic variants in known aberrated genes. Disease-causing genes are the most reliable pathogenic molecules with greatest impact on the development, course and prognosis of the disease. Identification of these genes has led to an increased understanding of disease pathogenesis and identification of druggable seeds, promoting research of rare and undiagnosed diseases, common diseases, and human physiology. Furthermore, the IRUD has greatly contributed to genomic medicine by identifying a large number of novel pathogenic variants in known aberrated genes. Approximately two-thirds of the identified pathogenic variants were novel, supporting the indispensable role of the IRUD in genomic medicine in addition to its diagnostic services. Aggregation of phenotypic and pathogenic variant data can enrich variant databases such as ClinVar (https://www.ncbi.nlm.nih.gov/clinvar/), LOVD (https://www.lovd.nl/), and MGeND (https://mgend.med.kyoto-u.ac.jp/) as well as disease databases such as OMIM and Orphanet (https://www.orpha.net/).

A remarkable feature of the IRUD is the extensive data sharing through the IRUD Exchange, which has accumulated HPO-based phenotypic and genetic data to enable searches for similar cases automatically by pattern-matching algorithms and solving N-of-1 problems. The IRUD Exchange is compatible with MME and functions as a gateway to domestic and international collaborative networks. The IRUD is one of the principal eight nodes of international genomic research projects connected via the MME (https://www.matchmakerexchange.org/participants.html). The collaboration network provides a collective dataset spanning more than 150,000 cases from more than 11,000 contributors in 88 countries [48]. Thus, the IRUD greatly contributes to international data sharing.

The IRUD-CL/SCL plays an essential role in covering broad aspects of genomic medicine as the only contact site for patients, where the IRUD-DB conducts pre-entry and post-analysis evaluation; establishes a final clinical diagnosis; and offers opportunities for genetic counseling, patient follow-up, public relations, regional cooperation, transitional medicine, and human resource development. Thus, the IRUD-CL/SCL is a clinical core facility not only in genomic medicine but also in medical systems for rare and intractable diseases, Nan-byo. Moreover, IRUD-DB has substantially improved both the clinical and research levels of IRUD in a cost-effective manner. Cost-effectiveness has been achieved by utilizing the diagnostic process of the national healthcare insurance system in Japan so that pre-screening for IRUD entry is covered by the system, enabling funding for the IRUD to be concentrated on research. Therefore, IRUD-DBs play a major role on both the quality and cost-effectiveness by conducting thorough pre-entry investigation within the health care system to maintain the optimum standard for IRUD entry best-suited for comprehensive genomic analysis and discovery of novel disease genes/entities.

Finally, IRUD has promoted human resource development for genome medicine or research and for rare diseases (Nan-byo). Activities in the IRUD encompass entire fields of genome medicine or research, including phenotyping, genetic analysis, informatics, diagnosis, and counseling. Experiences in the IRUD would be of great help in individual career development. The IRUD also provides excellent educational opportunities for medical geneticists, genetic counselors, and data scientists. Therefore, the IRUD is not only a nationwide diagnostic and research system, but also a sustainable human resource development system in Japan.

Notwithstanding the exhaustive WES, nearly 3000 pedigrees remained undiagnosed. Particularly, 313 pedigrees belonged to the N-of-1 category, for which discovery of another pedigree with the same candidate genes should be definitely necessary. Further promotion of international collaboration is the key to address the N-of-1 issue. On top of that, reasons for the undetermined causes are thought to include mosaicism, genomic alterations, gene regulation, and complex inheritance, all of which are difficult to capture by WES [49]. To address these issues, the IRUD has begun to adopt the cutting-edge strategies of WGS, long-read sequencing, RNA sequencing, epigenetics, proteomics, and metabolomics analysis. IRUD is one of the leading projects of the National Execution Plan for WGS, a national project promoting WGS-based genomic medicine. Furthermore, IRUD Beyond has been launched to provide a prioritized opportunity for IRUD to conduct functional studies using animal models and therapeutic studies using induced pluripotent stem cells or genome editing [50]. Taken together, IRUD continues to move forward until carrying out its mission to determine causes and provide cures for all the rare and undiagnosed diseases.

## Supplementary information

List of genes and number of novel and known pathogenic variants

Novel genes and phenotypes

## Data Availability

All the data and materials are available for academia, researchers or private enterprises, either domestic or abroad, upon examination and permission of usage proposals by IRUD Promotion Board (IRUD-PB) and CEC. Usage for profitable researches is restricted to those which contribute to the progress of medical fields. The sequence data of known pathogenic variants are available in Medical Genomics Japan Variant Database (MGeND) (https://mgend.med.kyoto-u.ac.jp/) supported by AMED.
