# Supplementary material for: Six years’ accomplishment of the Initiative on Rare and Undiagnosed Diseases: nationwide project in Japan to discover causes, mechanisms, and cures
Source: J Hum Genet. 2022 Mar 23;67(9):505–13. doi: 10.1038/s10038-022-01025-0 (PMC9402437; doi:10.1038/s10038-022-01025-0)
Supplement: Supplementary file 1 — List of genes and number of novel and known pathogenic variants [file 10038_2022_1025_MOESM1_ESM.docx]

**Supplemental Table 1.** List of genes and number of novel and known pathogenic variants

| gene | known | novel | total |
| --- | --- | --- | --- |
| *CHD7* | 13 | 18 | 31 |
| *MEFV* | 26 | 1 | 27 |
| *ARID1B* | 3 | 22 | 25 |
| *PTPN11* | 17 | 7 | 24 |
| *FBN1* | 14 | 10 | 24 |
| *NF1* | 5 | 18 | 23 |
| *MECP2* | 9 | 13 | 22 |
| *ACVRL1* | 8 | 5 | 13 |
| *PTEN* | 3 | 10 | 13 |
| *CREBBP* | 7 | 5 | 12 |
| *KMT2D* | 6 | 6 | 12 |
| *KMT2A* | 3 | 9 | 12 |
| *BRAF* | 9 | 2 | 11 |
| *RYR1* | 7 | 4 | 11 |
| *ASXL3* | 1 | 10 | 11 |
| *FOXG1* | 3 | 7 | 10 |
| *SMC1A* | 2 | 8 | 10 |
| *COL2A1* | 3 | 6 | 9 |
| *GRIN2B* | 3 | 6 | 9 |
| *PTCH1* | 2 | 7 | 9 |
| *EP300* | 1 | 8 | 9 |
| *MAGEL2* | 1 | 8 | 9 |
| *PURA* | 1 | 8 | 9 |
| *COL3A1* | 5 | 3 | 8 |
| *SPAST* | 5 | 3 | 8 |
| *COL4A4* | 3 | 5 | 8 |
| *COL5A1* | 3 | 5 | 8 |
| *DDX3X* | 2 | 6 | 8 |
| *KAT6B* | 2 | 6 | 8 |
| *DNAH5* | 1 | 7 | 8 |
| *PPP1CB* | 7 | 0 | 7 |
| *ENG* | 5 | 2 | 7 |
| *ASXL1* | 4 | 3 | 7 |
| *KAT6A* | 4 | 3 | 7 |
| *PPP2R5D* | 4 | 3 | 7 |
| *ADNP* | 3 | 4 | 7 |
| *ATRX* | 3 | 4 | 7 |
| *HECW2* | 3 | 4 | 7 |
| *SCN1A* | 3 | 4 | 7 |
| *KIF1A* | 2 | 5 | 7 |
| *VPS13B* | 2 | 5 | 7 |
| *DNMT3A* | 1 | 6 | 7 |
| *MED13L* | 1 | 6 | 7 |
| *NSD1* | 1 | 6 | 7 |
| *AHDC1* | 0 | 7 | 7 |
| *NIPBL* | 0 | 7 | 7 |
| *FGFR2* | 6 | 0 | 6 |
| *CDKL5* | 5 | 1 | 6 |
| *RNF213* | 5 | 1 | 6 |
| *NTRK1* | 4 | 2 | 6 |
| *SATB2* | 4 | 2 | 6 |
| *C5orf42* | 3 | 3 | 6 |
| *CACNA1A* | 3 | 3 | 6 |
| *EDA* | 3 | 3 | 6 |
| *GNAO1* | 3 | 3 | 6 |
| *IARS2* | 3 | 3 | 6 |
| *KCNQ2* | 3 | 3 | 6 |
| *ALMS1* | 2 | 4 | 6 |
| *CTNNB1* | 2 | 4 | 6 |
| *IQSEC2* | 2 | 4 | 6 |
| *CEP152* | 1 | 5 | 6 |
| *COL4A1* | 1 | 5 | 6 |
| *NBAS* | 1 | 5 | 6 |
| *SACS* | 1 | 5 | 6 |
| *CSPP1* | 0 | 6 | 6 |
| *GRIN1* | 0 | 6 | 6 |
| *SCN9A* | 0 | 6 | 6 |
| *SLC4A1* | 5 | 0 | 5 |
| *CPLANE1* | 4 | 1 | 5 |
| *GUSB* | 4 | 1 | 5 |
| *HRAS* | 4 | 1 | 5 |
| *MTM1* | 3 | 2 | 5 |
| *PACS1* | 3 | 2 | 5 |
| *TWIST1* | 3 | 2 | 5 |
| *WDR45* | 3 | 2 | 5 |
| *CASK* | 2 | 3 | 5 |
| *CLCN5* | 2 | 3 | 5 |
| *HDAC8* | 2 | 3 | 5 |
| *PDHA1* | 2 | 3 | 5 |
| *ACTB* | 1 | 4 | 5 |
| *EFTUD2* | 1 | 4 | 5 |
| *JAG1* | 1 | 4 | 5 |
| *NALCN* | 1 | 4 | 5 |
| *TAB2* | 1 | 4 | 5 |
| *ANKRD11* | 0 | 5 | 5 |
| *EBF3* | 4 | 0 | 4 |
| *KRAS* | 4 | 0 | 4 |
| *SHOC2* | 4 | 0 | 4 |
| *ATP7B* | 3 | 1 | 4 |
| *CDC42* | 3 | 1 | 4 |
| *CHRNG* | 3 | 1 | 4 |
| *KLHL40* | 3 | 1 | 4 |
| *NOTCH3* | 3 | 1 | 4 |
| *SMAD4* | 3 | 1 | 4 |
| *SOS1* | 3 | 1 | 4 |
| *B3GALT6* | 2 | 2 | 4 |
| *CEP290* | 2 | 2 | 4 |
| *GLB1* | 2 | 2 | 4 |
| *OFD1* | 2 | 2 | 4 |
| *PAX6* | 2 | 2 | 4 |
| *TH* | 2 | 2 | 4 |
| *TUBA1A* | 2 | 2 | 4 |
| *ALDH7A1* | 1 | 3 | 4 |
| *COL1A1* | 1 | 3 | 4 |
| *CSNK2A1* | 1 | 3 | 4 |
| *DYRK1A* | 1 | 3 | 4 |
| *KCNT1* | 1 | 3 | 4 |
| *KDM6A* | 1 | 3 | 4 |
| *PPP2R1A* | 1 | 3 | 4 |
| *SMARCA4* | 1 | 3 | 4 |
| *ATP7A* | 0 | 4 | 4 |
| *AUTS2* | 0 | 4 | 4 |
| *DYNC2H1* | 0 | 4 | 4 |
| *HSPG2* | 0 | 4 | 4 |
| *KARS* | 0 | 4 | 4 |
| *KDM5C* | 0 | 4 | 4 |
| *LARS* | 0 | 4 | 4 |
| *PIK3CA* | 0 | 4 | 4 |
| *POLR3A* | 0 | 4 | 4 |
| *RPS6KA3* | 0 | 4 | 4 |
| *TRIO* | 0 | 4 | 4 |
| *UBE3A* | 0 | 4 | 4 |
| *USP9X* | 0 | 4 | 4 |
| *ZBTB20* | 0 | 4 | 4 |
| *ZC4H2* | 0 | 4 | 4 |
| *MSH2* | 3 | 0 | 3 |
| *SCN5A* | 3 | 0 | 3 |
| *ATP1A3* | 2 | 1 | 3 |
| *BICD2* | 2 | 1 | 3 |
| *CAV3* | 2 | 1 | 3 |
| *CHST14* | 2 | 1 | 3 |
| *POLD1* | 2 | 1 | 3 |
| *RRAS2* | 2 | 1 | 3 |
| *TGFBR2* | 2 | 1 | 3 |
| *AKT3* | 1 | 2 | 3 |
| *ALDH3A2* | 1 | 2 | 3 |
| *APC* | 1 | 2 | 3 |
| *ASPM* | 1 | 2 | 3 |
| *CBL* | 1 | 2 | 3 |
| *CDK13* | 1 | 2 | 3 |
| *COL11A2* | 1 | 2 | 3 |
| *COL4A5* | 1 | 2 | 3 |
| *COMP* | 1 | 2 | 3 |
| *FBN2* | 1 | 2 | 3 |
| *FH* | 1 | 2 | 3 |
| *FLNA* | 1 | 2 | 3 |
| *GRIN2A* | 1 | 2 | 3 |
| *KMT2B* | 1 | 2 | 3 |
| *MED12* | 1 | 2 | 3 |
| *NRAS* | 1 | 2 | 3 |
| *PLA2G6* | 1 | 2 | 3 |
| *RARB* | 1 | 2 | 3 |
| *RERE* | 1 | 2 | 3 |
| *SLCO2A1* | 1 | 2 | 3 |
| *SPINK1* | 1 | 2 | 3 |
| *SRCAP* | 1 | 2 | 3 |
| *SYNGAP1* | 1 | 2 | 3 |
| *TSC2* | 1 | 2 | 3 |
| *ACTA1* | 0 | 3 | 3 |
| *ARID1A* | 0 | 3 | 3 |
| *BCOR* | 0 | 3 | 3 |
| *CLTC* | 0 | 3 | 3 |
| *CNOT3* | 0 | 3 | 3 |
| *COL11A1* | 0 | 3 | 3 |
| *CSNK2B* | 0 | 3 | 3 |
| *EDARADD* | 0 | 3 | 3 |
| *ELN* | 0 | 3 | 3 |
| *GATAD2B* | 0 | 3 | 3 |
| *GCK* | 0 | 3 | 3 |
| *GLI3* | 0 | 3 | 3 |
| *GPC3* | 0 | 3 | 3 |
| *HUWE1* | 0 | 3 | 3 |
| *MAP3K7* | 0 | 3 | 3 |
| *MID1* | 0 | 3 | 3 |
| *MORC2* | 0 | 3 | 3 |
| *MYH3* | 0 | 3 | 3 |
| *NEB* | 0 | 3 | 3 |
| *PCNT* | 0 | 3 | 3 |
| *PUF60* | 0 | 3 | 3 |
| *RECQL4* | 0 | 3 | 3 |
| *SALL4* | 0 | 3 | 3 |
| *SHANK3* | 0 | 3 | 3 |
| *SLC9A6* | 0 | 3 | 3 |
| *SMARCA2* | 0 | 3 | 3 |
| *SON* | 0 | 3 | 3 |
| *TBL1XR1* | 0 | 3 | 3 |
| *TCF4* | 0 | 3 | 3 |
| *TRRAP* | 0 | 3 | 3 |
| *CANT1* | 2 | 0 | 2 |
| *CPT2* | 2 | 0 | 2 |
| *CTBP1* | 2 | 0 | 2 |
| *DCX* | 2 | 0 | 2 |
| *DUOX2* | 2 | 0 | 2 |
| *EYS* | 2 | 0 | 2 |
| *GFAP* | 2 | 0 | 2 |
| *GJB1* | 2 | 0 | 2 |
| *GNB1* | 2 | 0 | 2 |
| *HIST1H1E* | 2 | 0 | 2 |
| *IGHMBP2* | 2 | 0 | 2 |
| *MPV17* | 2 | 0 | 2 |
| *NT5E* | 2 | 0 | 2 |
| *OTC* | 2 | 0 | 2 |
| *RAF1* | 2 | 0 | 2 |
| *SKI* | 2 | 0 | 2 |
| *SPTBN2* | 2 | 0 | 2 |
| *ST3GAL5* | 2 | 0 | 2 |
| *TMEM173* | 2 | 0 | 2 |
| *TNFRSF1A* | 2 | 0 | 2 |
| *TPM2* | 2 | 0 | 2 |
| *TYR* | 2 | 0 | 2 |
| *ABCG5* | 1 | 1 | 2 |
| *ACSF3* | 1 | 1 | 2 |
| *ACTA2* | 1 | 1 | 2 |
| *ADGRV1* | 1 | 1 | 2 |
| *AMER1* | 1 | 1 | 2 |
| *AP4S1* | 1 | 1 | 2 |
| *ARSA* | 1 | 1 | 2 |
| *ATM* | 1 | 1 | 2 |
| *ATP6V0A2* | 1 | 1 | 2 |
| *BTK* | 1 | 1 | 2 |
| *CBS* | 1 | 1 | 2 |
| *CDK19* | 1 | 1 | 2 |
| *CHD8* | 1 | 1 | 2 |
| *COL5A2* | 1 | 1 | 2 |
| *COL6A1* | 1 | 1 | 2 |
| *COL6A2* | 1 | 1 | 2 |
| *COL6A3* | 1 | 1 | 2 |
| *DHDDS* | 1 | 1 | 2 |
| *DNM1L* | 1 | 1 | 2 |
| *DYSF* | 1 | 1 | 2 |
| *ERF* | 1 | 1 | 2 |
| *FGFR1* | 1 | 1 | 2 |
| *FLNB* | 1 | 1 | 2 |
| *FOXC1* | 1 | 1 | 2 |
| *IFT172* | 1 | 1 | 2 |
| *IRF2BPL* | 1 | 1 | 2 |
| *KCNH2* | 1 | 1 | 2 |
| *KIF7* | 1 | 1 | 2 |
| *LIPH* | 1 | 1 | 2 |
| *LTBP4* | 1 | 1 | 2 |
| *MAP2K1* | 1 | 1 | 2 |
| *MYH7* | 1 | 1 | 2 |
| *NEBL* | 1 | 1 | 2 |
| *NFIX* | 1 | 1 | 2 |
| *OTUD7A* | 1 | 1 | 2 |
| *PIEZO1* | 1 | 1 | 2 |
| *PYGM* | 1 | 1 | 2 |
| *RAC1* | 1 | 1 | 2 |
| *RET* | 1 | 1 | 2 |
| *SCN2A* | 1 | 1 | 2 |
| *SCN4A* | 1 | 1 | 2 |
| *SCN8A* | 1 | 1 | 2 |
| *SGSH* | 1 | 1 | 2 |
| *SLC22A12* | 1 | 1 | 2 |
| *SPTAN1* | 1 | 1 | 2 |
| *TBC1D24* | 1 | 1 | 2 |
| *TGM1* | 1 | 1 | 2 |
| *TP63* | 1 | 1 | 2 |
| *TRAF7* | 1 | 1 | 2 |
| *TUBB* | 1 | 1 | 2 |
| *UNC13D* | 1 | 1 | 2 |
| *ZMYND11* | 1 | 1 | 2 |
| *AARS2* | 0 | 2 | 2 |
| *ABCA12* | 0 | 2 | 2 |
| *ABCG8* | 0 | 2 | 2 |
| *ACE* | 0 | 2 | 2 |
| *ACO2* | 0 | 2 | 2 |
| *ACTL6B* | 0 | 2 | 2 |
| *ALG9* | 0 | 2 | 2 |
| *AP3B1* | 0 | 2 | 2 |
| *APTX* | 0 | 2 | 2 |
| *BBS10* | 0 | 2 | 2 |
| *BBS7* | 0 | 2 | 2 |
| *BRF1* | 0 | 2 | 2 |
| *C3orf17* | 0 | 2 | 2 |
| *CAPN3* | 0 | 2 | 2 |
| *CDK8* | 0 | 2 | 2 |
| *CHST3* | 0 | 2 | 2 |
| *CLN8* | 0 | 2 | 2 |
| *COL1A2* | 0 | 2 | 2 |
| *CTNS* | 0 | 2 | 2 |
| *CUL4B* | 0 | 2 | 2 |
| *CYP17A1* | 0 | 2 | 2 |
| *DDX59* | 0 | 2 | 2 |
| *DLG3* | 0 | 2 | 2 |
| *DNAH1* | 0 | 2 | 2 |
| *DSCAM* | 0 | 2 | 2 |
| *EIF2S3* | 0 | 2 | 2 |
| *ERCC2* | 0 | 2 | 2 |
| *ERLIN2* | 0 | 2 | 2 |
| *FAS* | 0 | 2 | 2 |
| *FBXL4* | 0 | 2 | 2 |
| *FLNC* | 0 | 2 | 2 |
| *FOXP1* | 0 | 2 | 2 |
| *GATA2* | 0 | 2 | 2 |
| *GBA2* | 0 | 2 | 2 |
| *GPD1* | 0 | 2 | 2 |
| *GRIA2* | 0 | 2 | 2 |
| *HKDC1* | 0 | 2 | 2 |
| *HNF1A* | 0 | 2 | 2 |
| *HNRNPU* | 0 | 2 | 2 |
| *IGLL1* | 0 | 2 | 2 |
| *KIAA0586* | 0 | 2 | 2 |
| *KIAA0753* | 0 | 2 | 2 |
| *KIF11* | 0 | 2 | 2 |
| *KIF6* | 0 | 2 | 2 |
| *KMT2C* | 0 | 2 | 2 |
| *LARS2* | 0 | 2 | 2 |
| *LIG3* | 0 | 2 | 2 |
| *LSR* | 0 | 2 | 2 |
| *LZTR1* | 0 | 2 | 2 |
| *MAN1B1* | 0 | 2 | 2 |
| *MAPK8IP3* | 0 | 2 | 2 |
| *MARS* | 0 | 2 | 2 |
| *MEIS2* | 0 | 2 | 2 |
| *MITF* | 0 | 2 | 2 |
| *MOCS1* | 0 | 2 | 2 |
| *MTPAP* | 0 | 2 | 2 |
| *MYBPC3* | 0 | 2 | 2 |
| *NAGLU* | 0 | 2 | 2 |
| *NOD2* | 0 | 2 | 2 |
| *NPC1* | 0 | 2 | 2 |
| *NR2F1* | 0 | 2 | 2 |
| *OOEP* | 0 | 2 | 2 |
| *PDHX* | 0 | 2 | 2 |
| *PHEX* | 0 | 2 | 2 |
| *PHKA2* | 0 | 2 | 2 |
| *PIGW* | 0 | 2 | 2 |
| *POGZ* | 0 | 2 | 2 |
| *POLR2A* | 0 | 2 | 2 |
| *POMT2* | 0 | 2 | 2 |
| *RASA1* | 0 | 2 | 2 |
| *RNPC3* | 0 | 2 | 2 |
| *RTEL1* | 0 | 2 | 2 |
| *SALL1* | 0 | 2 | 2 |
| *SETD2* | 0 | 2 | 2 |
| *SGCE* | 0 | 2 | 2 |
| *SLC13A5* | 0 | 2 | 2 |
| *SMAD3* | 0 | 2 | 2 |
| *SMS* | 0 | 2 | 2 |
| *SOX2* | 0 | 2 | 2 |
| *SOX5* | 0 | 2 | 2 |
| *SPINK5* | 0 | 2 | 2 |
| *SPR* | 0 | 2 | 2 |
| *STAG2* | 0 | 2 | 2 |
| *STXBP1* | 0 | 2 | 2 |
| *SYNJ1* | 0 | 2 | 2 |
| *TMEM67* | 0 | 2 | 2 |
| *TNFAIP3* | 0 | 2 | 2 |
| *TNXB* | 0 | 2 | 2 |
| *TRIT1* | 0 | 2 | 2 |
| *TRMT1* | 0 | 2 | 2 |
| *TUBB4A* | 0 | 2 | 2 |
| *TUBGCP6* | 0 | 2 | 2 |
| *USP7* | 0 | 2 | 2 |
| *VHL* | 0 | 2 | 2 |
| *VIPAS39* | 0 | 2 | 2 |
| *WFS1* | 0 | 2 | 2 |
| *ZEB2* | 0 | 2 | 2 |
| *AGXT* | 1 | 0 | 1 |
| *AKT1* | 1 | 0 | 1 |
| *ALG11* | 1 | 0 | 1 |
| *ANKH* | 1 | 0 | 1 |
| *AR* | 1 | 0 | 1 |
| *ARF1* | 1 | 0 | 1 |
| *ARSE* | 1 | 0 | 1 |
| *ASPA* | 1 | 0 | 1 |
| *ATAD3A* | 1 | 0 | 1 |
| *ATP2A2* | 1 | 0 | 1 |
| *ATP6V1B2* | 1 | 0 | 1 |
| *BAG3* | 1 | 0 | 1 |
| *BBS1* | 1 | 0 | 1 |
| *BMPR2* | 1 | 0 | 1 |
| *C12orf65* | 1 | 0 | 1 |
| *CACNA1S* | 1 | 0 | 1 |
| *CAMK2A* | 1 | 0 | 1 |
| *CAMK2B* | 1 | 0 | 1 |
| *CDKN1C* | 1 | 0 | 1 |
| *CFB* | 1 | 0 | 1 |
| *CHD2* | 1 | 0 | 1 |
| *CLCN1* | 1 | 0 | 1 |
| *CLCNKB* | 1 | 0 | 1 |
| *CNOT2* | 1 | 0 | 1 |
| *COL7A1* | 1 | 0 | 1 |
| *COL9A2* | 1 | 0 | 1 |
| *CRYAB* | 1 | 0 | 1 |
| *CSF1R* | 1 | 0 | 1 |
| *CTRC* | 1 | 0 | 1 |
| *DCDC2* | 1 | 0 | 1 |
| *DMD* | 1 | 0 | 1 |
| *DPF2* | 1 | 0 | 1 |
| *EDN3* | 1 | 0 | 1 |
| *EFNB1* | 1 | 0 | 1 |
| *EIF2B2* | 1 | 0 | 1 |
| *ESPN* | 1 | 0 | 1 |
| *EYA1* | 1 | 0 | 1 |
| *FGF12* | 1 | 0 | 1 |
| *FGG* | 1 | 0 | 1 |
| *FKTN* | 1 | 0 | 1 |
| *FRMD7* | 1 | 0 | 1 |
| *GABRA1* | 1 | 0 | 1 |
| *GABRB3* | 1 | 0 | 1 |
| *GATA6* | 1 | 0 | 1 |
| *GJA8* | 1 | 0 | 1 |
| *GRIA3* | 1 | 0 | 1 |
| *HNF4A* | 1 | 0 | 1 |
| *IL10RA* | 1 | 0 | 1 |
| *KCND3* | 1 | 0 | 1 |
| *KCNQ3* | 1 | 0 | 1 |
| *KIAA2022* | 1 | 0 | 1 |
| *KIF5C* | 1 | 0 | 1 |
| *KVLQT1* | 1 | 0 | 1 |
| *KY* | 1 | 0 | 1 |
| *L1CAM* | 1 | 0 | 1 |
| *LDLR* | 1 | 0 | 1 |
| *LMNA* | 1 | 0 | 1 |
| *MAP1B* | 1 | 0 | 1 |
| *MAP2K2* | 1 | 0 | 1 |
| *MCM8* | 1 | 0 | 1 |
| *MED13* | 1 | 0 | 1 |
| *MEN1* | 1 | 0 | 1 |
| *MPZ* | 1 | 0 | 1 |
| *MSH6* | 1 | 0 | 1 |
| *MYH2* | 1 | 0 | 1 |
| *MYL2* | 1 | 0 | 1 |
| *OGT* | 1 | 0 | 1 |
| *PAX3* | 1 | 0 | 1 |
| *PBX1* | 1 | 0 | 1 |
| *PIEZO2* | 1 | 0 | 1 |
| *PIK3R2* | 1 | 0 | 1 |
| *PKP2* | 1 | 0 | 1 |
| *PLCB4* | 1 | 0 | 1 |
| *PLP1* | 1 | 0 | 1 |
| *PQBP1* | 1 | 0 | 1 |
| *PROC* | 1 | 0 | 1 |
| *PRRT2* | 1 | 0 | 1 |
| *RANBP2* | 1 | 0 | 1 |
| *RPS19* | 1 | 0 | 1 |
| *RRM2B* | 1 | 0 | 1 |
| *SCN1B* | 1 | 0 | 1 |
| *SERPINA7* | 1 | 0 | 1 |
| *SLC16A2* | 1 | 0 | 1 |
| *SLC25A13* | 1 | 0 | 1 |
| *SLC25A4* | 1 | 0 | 1 |
| *SLC26A2* | 1 | 0 | 1 |
| *SLC2A1* | 1 | 0 | 1 |
| *SLC33A1* | 1 | 0 | 1 |
| *SMARCB1* | 1 | 0 | 1 |
| *SOD1* | 1 | 0 | 1 |
| *TAZ* | 1 | 0 | 1 |
| *TFAP2A* | 1 | 0 | 1 |
| *TFE3* | 1 | 0 | 1 |
| *TFG* | 1 | 0 | 1 |
| *TGFB1* | 1 | 0 | 1 |
| *TGFBR1* | 1 | 0 | 1 |
| *TMEM107* | 1 | 0 | 1 |
| *TRPC6* | 1 | 0 | 1 |
| *TRPV4* | 1 | 0 | 1 |
| *TSHR* | 1 | 0 | 1 |
| *UBTF* | 1 | 0 | 1 |
| *WAS* | 1 | 0 | 1 |
| *WASF1* | 1 | 0 | 1 |
| *WT1* | 1 | 0 | 1 |
| *ZSWIM6* | 1 | 0 | 1 |
| *ABCA3* | 0 | 1 | 1 |
| *ABCB4* | 0 | 1 | 1 |
| *ACAN* | 0 | 1 | 1 |
| *ACTG1* | 0 | 1 | 1 |
| *ADAM10* | 0 | 1 | 1 |
| *ADAT3* | 0 | 1 | 1 |
| *AIFM1* | 0 | 1 | 1 |
| *ALPL* | 0 | 1 | 1 |
| *ANK1* | 0 | 1 | 1 |
| *ANO5* | 0 | 1 | 1 |
| *ANTXR2* | 0 | 1 | 1 |
| *ARCN1* | 0 | 1 | 1 |
| *ARF3* | 0 | 1 | 1 |
| *ARID2* | 0 | 1 | 1 |
| *ARX* | 0 | 1 | 1 |
| *ATL1* | 0 | 1 | 1 |
| *ATP6V0A4* | 0 | 1 | 1 |
| *ATP6V1B1* | 0 | 1 | 1 |
| *B4GALNT1* | 0 | 1 | 1 |
| *BBS4* | 0 | 1 | 1 |
| *BPTF* | 0 | 1 | 1 |
| *BRCA1* | 0 | 1 | 1 |
| *BRWD3* | 0 | 1 | 1 |
| *C12orf57* | 0 | 1 | 1 |
| *CCND2* | 0 | 1 | 1 |
| *CHD3* | 0 | 1 | 1 |
| *CHD4* | 0 | 1 | 1 |
| *CHRDL1* | 0 | 1 | 1 |
| *CHRNB2* | 0 | 1 | 1 |
| *CIC* | 0 | 1 | 1 |
| *CLCN2* | 0 | 1 | 1 |
| *COA7* | 0 | 1 | 1 |
| *COL11A* | 0 | 1 | 1 |
| *COL4A2* | 0 | 1 | 1 |
| *CPOX* | 0 | 1 | 1 |
| *CTNND2* | 0 | 1 | 1 |
| *CUL3* | 0 | 1 | 1 |
| *CYFIP2* | 0 | 1 | 1 |
| *DHX30* | 0 | 1 | 1 |
| *DLG4* | 0 | 1 | 1 |
| *DNASE1* | 0 | 1 | 1 |
| *DNMT1* | 0 | 1 | 1 |
| *DTNB* | 0 | 1 | 1 |
| *DVL1* | 0 | 1 | 1 |
| *DYNC1H1* | 0 | 1 | 1 |
| *EDNRB* | 0 | 1 | 1 |
| *EHMT1* | 0 | 1 | 1 |
| *EHMT2* | 0 | 1 | 1 |
| *EMHT1* | 0 | 1 | 1 |
| *ERCC8* | 0 | 1 | 1 |
| *ETV6* | 0 | 1 | 1 |
| *EXT1* | 0 | 1 | 1 |
| *EXT2* | 0 | 1 | 1 |
| *F2* | 0 | 1 | 1 |
| *FAM126A* | 0 | 1 | 1 |
| *FGD1* | 0 | 1 | 1 |
| *FGFR3* | 0 | 1 | 1 |
| *FLCN* | 0 | 1 | 1 |
| *FOXF1* | 0 | 1 | 1 |
| *FUK* | 0 | 1 | 1 |
| *FZD4* | 0 | 1 | 1 |
| *GCH1* | 0 | 1 | 1 |
| *GJA1* | 0 | 1 | 1 |
| *GLRA1* | 0 | 1 | 1 |
| *GMNN* | 0 | 1 | 1 |
| *GNAS* | 0 | 1 | 1 |
| *GNPTAB* | 0 | 1 | 1 |
| *GRHL2* | 0 | 1 | 1 |
| *GUCA1B* | 0 | 1 | 1 |
| *HDAC4* | 0 | 1 | 1 |
| *IFIH1* | 0 | 1 | 1 |
| *IGSF1* | 0 | 1 | 1 |
| *IKBKE* | 0 | 1 | 1 |
| *ITPR1* | 0 | 1 | 1 |
| *KANSL1* | 0 | 1 | 1 |
| *KCNB1* | 0 | 1 | 1 |
| *KCNK9* | 0 | 1 | 1 |
| *KCNMA1* | 0 | 1 | 1 |
| *KCNQ1* | 0 | 1 | 1 |
| *KDM2B* | 0 | 1 | 1 |
| *KIF21A* | 0 | 1 | 1 |
| *KMT2E* | 0 | 1 | 1 |
| *KRIT1* | 0 | 1 | 1 |
| *KRT14* | 0 | 1 | 1 |
| *LDB3* | 0 | 1 | 1 |
| *LRP5* | 0 | 1 | 1 |
| *MBD5* | 0 | 1 | 1 |
| *MECOM* | 0 | 1 | 1 |
| *MECP* | 0 | 1 | 1 |
| *MEF2C* | 0 | 1 | 1 |
| *MIEF2* | 0 | 1 | 1 |
| *MKKS* | 0 | 1 | 1 |
| *MMP2* | 0 | 1 | 1 |
| *MSL3* | 0 | 1 | 1 |
| *MT-ATP6* | 0 | 1 | 1 |
| *MTOR* | 0 | 1 | 1 |
| *MYCN* | 0 | 1 | 1 |
| *MYH10* | 0 | 1 | 1 |
| *MYH6* | 0 | 1 | 1 |
| *MYH9* | 0 | 1 | 1 |
| *NARS2* | 0 | 1 | 1 |
| *NCOA6* | 0 | 1 | 1 |
| *NCOR1* | 0 | 1 | 1 |
| *NFIA* | 0 | 1 | 1 |
| *NFKB1* | 0 | 1 | 1 |
| *NIPA1* | 0 | 1 | 1 |
| *NKX2-1* | 0 | 1 | 1 |
| *NLRC4* | 0 | 1 | 1 |
| *NLRP12* | 0 | 1 | 1 |
| *NONO* | 0 | 1 | 1 |
| *NOTCH1* | 0 | 1 | 1 |
| *NOTCH2* | 0 | 1 | 1 |
| *NPR2* | 0 | 1 | 1 |
| *NR1H4* | 0 | 1 | 1 |
| *NRXN2* | 0 | 1 | 1 |
| *NSD2* | 0 | 1 | 1 |
| *NSF* | 0 | 1 | 1 |
| *OPHN1* | 0 | 1 | 1 |
| *OTUD5* | 0 | 1 | 1 |
| *PACS2* | 0 | 1 | 1 |
| *PARN* | 0 | 1 | 1 |
| *PAX9* | 0 | 1 | 1 |
| *PHF6* | 0 | 1 | 1 |
| *PHOX2B* | 0 | 1 | 1 |
| *PIK3R1* | 0 | 1 | 1 |
| *PITX2* | 0 | 1 | 1 |
| *PKD1* | 0 | 1 | 1 |
| *PORCN* | 0 | 1 | 1 |
| *PPM1D* | 0 | 1 | 1 |
| *PPP3CA* | 0 | 1 | 1 |
| *PPRS1* | 0 | 1 | 1 |
| *PRG4* | 0 | 1 | 1 |
| *PTCHD1* | 0 | 1 | 1 |
| *PTH1R* | 0 | 1 | 1 |
| *PYCR2* | 0 | 1 | 1 |
| *RAD21* | 0 | 1 | 1 |
| *RAD51* | 0 | 1 | 1 |
| *RAI1* | 0 | 1 | 1 |
| *RBCK1* | 0 | 1 | 1 |
| *RBM10* | 0 | 1 | 1 |
| *RBM42* | 0 | 1 | 1 |
| *RHOBTB2* | 0 | 1 | 1 |
| *RORA* | 0 | 1 | 1 |
| *RP1L1* | 0 | 1 | 1 |
| *RPL13* | 0 | 1 | 1 |
| *RUNX2* | 0 | 1 | 1 |
| *RYR3* | 0 | 1 | 1 |
| *SASH1* | 0 | 1 | 1 |
| *SBDS* | 0 | 1 | 1 |
| *SCN11A* | 0 | 1 | 1 |
| *SETBP1* | 0 | 1 | 1 |
| *SETD5* | 0 | 1 | 1 |
| *SETX* | 0 | 1 | 1 |
| *SFTPA2* | 0 | 1 | 1 |
| *SIN3A* | 0 | 1 | 1 |
| *SIX1* | 0 | 1 | 1 |
| *SLAIN2* | 0 | 1 | 1 |
| *SLC1A2* | 0 | 1 | 1 |
| *SLC34A3* | 0 | 1 | 1 |
| *SMC3* | 0 | 1 | 1 |
| *SMPD4* | 0 | 1 | 1 |
| *SOX4* | 0 | 1 | 1 |
| *SOX9* | 0 | 1 | 1 |
| *SPTB* | 0 | 1 | 1 |
| *SPTBN4* | 0 | 1 | 1 |
| *STAG1* | 0 | 1 | 1 |
| *STAT3* | 0 | 1 | 1 |
| *SYNE1* | 0 | 1 | 1 |
| *SYT1* | 0 | 1 | 1 |
| *SZT2* | 0 | 1 | 1 |
| *TAF1* | 0 | 1 | 1 |
| *TBR1* | 0 | 1 | 1 |
| *TBX1* | 0 | 1 | 1 |
| *TBX5* | 0 | 1 | 1 |
| *TCF20* | 0 | 1 | 1 |
| *TLK2* | 0 | 1 | 1 |
| *TMED1* | 0 | 1 | 1 |
| *TNFRSF13B* | 0 | 1 | 1 |
| *TOR1AIP1* | 0 | 1 | 1 |
| *TRIP12* | 0 | 1 | 1 |
| *TUBB2A* | 0 | 1 | 1 |
| *TUBB3* | 0 | 1 | 1 |
| *UBAP1* | 0 | 1 | 1 |
| *UBE2A* | 0 | 1 | 1 |
| *UPF1* | 0 | 1 | 1 |
| *USH2A* | 0 | 1 | 1 |
| *VAV1* | 0 | 1 | 1 |
| *WAC* | 0 | 1 | 1 |
| *WDFY3* | 0 | 1 | 1 |
| *WDR26* | 0 | 1 | 1 |
| *ZBTB18* | 0 | 1 | 1 |
| *ZDHHC9* | 0 | 1 | 1 |
| *ZIC1* | 0 | 1 | 1 |
| Total (657 genes) | 605 | 1113 | 1718 |
