# Supplementary material for: Six years’ accomplishment of the Initiative on Rare and Undiagnosed Diseases: nationwide project in Japan to discover causes, mechanisms, and cures
Source: J Hum Genet. 2022 Mar 23;67(9):505–13. doi: 10.1038/s10038-022-01025-0 (PMC9402437; doi:10.1038/s10038-022-01025-0)
Supplement: Supplementary file 2 — Novel genes and phenotypes [file 10038_2022_1025_MOESM2_ESM.docx]

**Category 1:** Novel disease entities with novel aberrated genes

| **Disease entity** | **OMIM ID** | **Gene** | **Reference** |
| --- | --- | --- | --- |
| Radioulnar synostosis with amegakaryocytic thrombocytopenia 2 | 616738 | *EVI1* | Niihori T et al. 2015 (ref. 13) |
| Takenouchi-Kosaki syndrome | 616737 | *CDC42* | Takenouchi T. et al. 2015 (ref. 14) |
| ZTTK syndrome | 617140 | *SON* | Takenouchi T et al, 2016 (ref. 15) |
| Mental retardation, autosomal recessive 53 | 616917 | *PIGG* | Makrythanasis P et al. 2016 (ref. 16) |
| Pontocerebellar hypoplasia, type 7 | 614969 | *TOE1* | Lardelli RM et al. 2017 (ref. 17) |
| Bosma arhinia microphthalmia syndrome | 603457 | *SMCHD1* | Shaw ND et al. 2017 (ref. 18) |
| Gabriele-de Vries syndrome | 617557 | *YY1* | Gabriele M et al., 2017 (ref. 19) |
| Steroid-responsive primary nephrosis | Not assigned | *ITSN2* | Ashraf S et al., 2018 (ref. 20) |
| Intellectual disability, epilepsy, and autism | Not assigned | *SETD1B* | Hiraide T et. al 2018 (ref. 21) |
| Galactosemia IV | 618881 | *GALM* | Wada Y et al. 2019 (ref. 22) |
| Neurodevelopmental disorder with or without variable brain abnormalities | 618443 | *MAPK8IP3* | Iwasawa S et al. 2019 (ref. 23) |
| Noonan syndrome 12 | 618624 | *RRAS2* | Niihori T et al. 2019 (ref. 24) |
| Intellectual developmental disorder with nasal speech, dysmorphic facies, and variable skeletal anomalies | 618608 | *CNOT2* | Uehara T et al, 2019 (ref. 25) |
| Early infantile epileptic encephalopathy | Not assigned | *NSF* | Suzuki H et al, 2019 (ref. 26) |
| Infantile intrahepatic cholestasis | Not assigned | *LSR* | Uehara T et al, 2020 (ref. 27) |
| Aplastic anemia, mental retardation, and dwarfism syndrome | 619151 | *ALDH2,ADH5* | Oka Y et al. 2020 (ref. 28) |
| Intellectual disability, mesomelic dysplasia, horseshoe kidney, and epileptic encephalopathy | Not assigned | *AFF3* | Voisin N et al. 2021 (ref. 29) |
| Neurodevelopmental and eye abnormalities | Not assigned | *PRR12* | Chowdhury F et al. 2021 (ref. 30) |
| Severe intellectual disability, hypotonia, and seizures | Not assigned | *OTUD7A* | Suzuki H et al. 2021 (ref. 31) |
| Developmental and epileptic encephalopathy 87 | 618916 | *CDK19* | Zarate YA et al. 2021 (ref. 32) |
| Not specified | *** | ***** | Manuscript in preparation |
| Not specified | *** | ***** | Manuscript in preparation |

**Category 2:** Novel disease entities (Unique OMIM IDs) with known aberrated genes

| **Disease Entity** | **OMIM ID** | **Gene** | **Reference** |
| --- | --- | --- | --- |
| Kosaki Overgrowth syndrome | 616592 | *PDGFRB* | Minatogawa M et al. 2017 (ref. 33) |
| Arthrogryposis, cleft palate, craniosynostosis, and impaired intellectual development | 618265 | *PPP3CA* | Mizuguchi T et al. 2018 (ref. 34) |
| Cornelia de Lange syndrome 2 | 300590 | *SMC1A* | Chinen et al., 2019 (ref. 35) |
| Noonan syndrome | 618499 | *MRAS* | Suzuki H et al. 2019 (ref. 36) |

**Category 3:** Novel phenotypes with known disease entities and aberrated genes

| **Disease entity** | **OMIM ID** | **Gene** | **Reference** |
| --- | --- | --- | --- |
| Hydranencephaly-like severe form of cortical dysgenesis (Lissencephaly 3) | 611603 | *TUBA1A* | Yokoi S et al. 2015 (ref. 37) |
| Overlap with Coffin–Siris Syndrome (Nicolaides-Baraitser syndrome) | 601358 | *SMARCA2* duplication | Miyake N et al. 2016 (ref. 38) |
| West syndrome (Neurodegeneration with brain iron accumulation 5) | 300894 | *WDR45* | Nakashima M et al. 2016 (ref. 39) |
| Schimmelpenning-Feuerstein-Mims syndrome (Noonan syndrome 7) | 613706 | *BRAF* | Watanabe Y et al. 2016 (ref. 40) |
| (Cole-Carpenter syndrome 2) | 616294 | *SEC24D* | Takeyari S et al. 2018 (ref. 41) |
| Severe apnea and sick sinus syndrome (encephalopathy, neonatal severe) | 300673 | *MECP2* | Shioda T et al. 2018 (ref. 42) |
| Early childhood-onset type 1B diabetes (maturity-onset diabetes of the young, type VII) | 610508 | *KLF11* | Ushijima K et al. 2019 (ref. 43) |
| Global developmental delay with seizures (Okur-Chung neurodevelopmental syndrome/Poirier-Bienvenu neurodevelopmental syndrome) | 617062/618732 | *CSNK2A1, CSNK2B* | Nakashima M et al. 2019 (ref. 44) |
| Oral-facial-digital syndrome (short-rib thoracic dysplasia 10 with or without polydactyly) | 615630 | *IFT172* | Yamada M et al. 2019 (ref. 45) |
| West syndrome (epileptic encephalopathy, early infantile, 47) | 617166 | *FGF12* duplication | Oda Y et al. 2019 (ref. 46) |
| Auriculocondylar syndrome 1 | 602483 | *GNAI3* | Yanagi K et al. 2021 (ref. 47) |
| Abnormality of the nervous system plus | *** | ***** | Manuscript in preparation |
